# Molecular Characterization and Prevalence of *Trypanozoon* Infection in Livestock on Small-Scale Farms in Thailand: An Integrative Control Approach

**DOI:** 10.3390/pathogens15080790

**Published:** 2026-07-24

**Authors:** Apiraya Rudeekiatthamrong, Pairpailin Jhaiaun, Giang Thi Nguyen, Wissanuwat Chimnoi, Darunwan Chuensaengarun, Chattraporn Rungchalermlak, Tanapat Mutchimadilok, Nipa Thammasonthijarern, Thawijit Phannithi, Tawanhathai Apichaimongkonkun, Laphatsararach Apinantanakorn, Kanittha Phetudomsinsuk, Tawin Inpankaew, Burin Nimsuphan, Ruttayaporn Ngasaman, Nuttapon Manojai, Jumnongjit Phasuk, Ketsarin Kamyingkird

**Affiliations:** 1 Department of Parasitology, Faculty of Veterinary Medicine, Kasetsart University, Lad Yao, Chatuchak, Bangkok 10900, Thailand; apiraya.rt@gmail.com (A.R.); pairpailin.j@gmail.com (P.J.); giangthi.n@ku.th (G.T.N.); wissanuwat.c@ku.th (W.C.); darunwan.chu@ku.th (D.C.); chattraporn.rung@ku.th (C.R.); tanapat.mut@ku.th (T.M.); nipa.tha@ku.th (N.T.); tawin.i@ku.th (T.I.); fvetbrn@ku.ac.th (B.N.); 2 Kasetsart University Animal Teaching Hospital, Faculty of Veterinary Medicine, Kasetsart University, Kamphaeng Saen, Nakorn Pathom 73140, Thailand; dugezia@hotmail.com (T.P.); bellkungvet71@gmail.com (T.A.); fvetruk@ku.ac.th (L.A.); 3 Department of Large Animal and Wildlife Clinical Sciences, Faculty of Veterinary Medicine, Kasetsart University, Kamphaeng Saen, Nakorn Pathom 73140, Thailand; kanittha.p@ku.th; 4 Faculty of Veterinary Science, Prince of Songkla University, Songkhla 90110, Thailand; ruttayaporn.n@psu.ac.th; 5 Mae Moh District Livestock Office, Mae Moh District, Lampang 52220, Thailand; nuttynitez@gmail.com

**Keywords:** *T. evansi* typing, molecular characterization, phylogenetic analysis, *Trypanozoon*, integrative control approach

## Abstract

Trypanosomosis (Surra), caused by the hemoflagellate protozoan *Trypanosoma evansi*, has a negative impact on animal health and the livestock economy in many countries, including Thailand. Molecular epidemiological data and integrative control approaches for small-scale farms remain limited. This study determined the prevalence of *Trypanozoon* infection and its associated risk factors in Thai livestock under an integrative control approach. Molecular tools were used to characterize *T. evansi* typing. A total of 947 blood samples were collected from cattle and buffalo across 123 small-scale farms in 16 provinces covering four regions of Thailand, with diagnostic results and control recommendations communicated to farmers within 14 days. The ITS2-positive samples were then subjected to further molecular characterization, together with two dogs, seven horses, and an in vitro *T. evansi* isolate, comprising 40 *Trypanozoon*-positive samples. These were characterized using PCR targeting the TBR primer, maxicircle NADH5, VSG RoTat 1.2, and minicircle B, followed by ITS2-based phylogenetic analysis. Risk factors were assessed using Chi-square and Fisher’s exact tests, logistic regression, a generalized linear mixed model, and Firth’s penalized logistic regression. The overall prevalence of *Trypanozoon* infection was 3.17% (30/947), detected exclusively in beef cattle in the Northern region (15.30%; 30/196), specifically in Lampang (46.15%, 12/26), Tak (23.08%, 3/13), and Phrae (17.24%, 15/87) Provinces. No infections were reported in the Northeastern, Central, or Southern regions. Male sex, age under one year, and residence in the Northern region were significant individual-level risk factors, while farm-level lacrimation and the presence of *Stomoxys* were significantly associated with infection. Molecular characterization identified *T. evansi* type A and type non-A/B, with no type B or maxicircle-positive samples detected. Phylogenetic analysis grouped the Thai isolates with previously reported isolates from Thai deer and cattle, Iranian camels, and Colombian dogs. On follow-up, no *Trypanozoon* infection was detected in revisited farms. These findings confirmed that *T. evansi*, comprising types A and non-A/B, continues to circulate in Thai livestock, particularly in beef cattle in Northern Thailand. Data from this study can be used for surveillance and future development of more effective diagnostic systems.

## 1. Introduction

*Trypanosoma evansi* (*T. evansi*) is a hemoflagellate protozoan parasite that causes trypanosomosis, commonly known as surra in animals [1,2]. This neglected tropical disease (NTD) has a significant economic and animal-health impact, especially in tropical and subtropical regions [3]. This parasite belongs to the subgenus *Trypanozoon*, which includes *T. brucei*, *T. rhodesiense*, *T. gambiense*, and *T. equiperdum* [4], and is a mutant derived from *T. brucei* that is lacking maxicircle kinetoplast DNA (kDNA) [5]. This prevents *T. evansi* from developing insect vectors as it cannot perform oxidative phosphorylation, which is essential for the insect’s midgut survival [6]. Thus, mechanical transmission via biting insects has become the predominant infection mode of this parasite, particularly in large domestic animals, including cattle, camels, horses, and water buffalo [1]. The geographical distribution of *T. evansi* covers Africa, Asia, and Latin America [7], infecting a wide range of mammalian species, including livestock (cattle, water buffalo, goats, horses) and wild or domestic animals (rodents, dogs, pigs, deer, rabbits) [8]. Human infections have also been reported in India and Vietnam [9,10]. 

Molecular techniques such as polymerase chain reaction (PCR) are commonly used to detect this parasite due to their high sensitivity and specificity. Several primers are available to detect and identify *Trypanosoma* species [11]. Detecting *Trypanosoma* DNA provides insight into the current infection in the animal. Data from these molecular methods are used to investigate the genetic diversity of *T. evansi*. The Manual of Diagnostic Tests and Vaccines for Terrestrial Animals from the World Organization for Animal Health (WOAH) states that the TBR primer is recommended as the gold standard for molecular diagnosis in the subgenus *Trypanozoon* [11]. The TBR primer amplifies a highly conserved satellite DNA sequence, which is found in the nucleus of the *Trypanozoon* subgenus [12]. Currently, three *T. evansi* types have been reported, including type A [13], type B [14], and type non-A/B [15]. The ILO7957/ILO8091 primer is used to detect *T. evansi* type A [13] and targets the variant surface glycoprotein (VSG) Rode *Trypanozoon* antigen type 1.2 (RoTat 1.2) gene target. This has become a useful tool for identifying *T. evansi* infection in endemic countries with mixed distributions of *Trypanosoma* spp. [11]. The VSG is an important structural component of trypanosomes, as a molecule that coats the parasite’s cell surface, functioning as an antigen. The VSG RoTat 1.2 is a predominant antigen in *T. evansi* but is absent in *T. brucei* [13]. *T. evansi* type B can be identified using the EVAB primer. This targets minicircle kDNA [14], which encodes guide RNAs for mitochondrial RNA editing and maintains the kinetoplast DNA structure in the mitochondrion [16]. The homogeneous minicircle kDNA in *T. evansi* is an interesting alternative candidate for developing useful markers to detect this parasite [14]. Recently, *T. evansi* type non-A/B has been reported in buffalo in Indonesia, which is negative for both the VSG RoTat 1.2 and minicircle B gene targets [15], making standard disease screening very difficult.

This finding is a new challenge in the diagnosis field to confirm *T. evansi* types. The diversity of *T. evansi* types also affects virulence, diagnostics, surveillance tools, and disease management, with several *T. evansi* isolates in livestock in the Philippines exhibiting high virulence and multiple drug resistance [17,18]. In Thailand, many blood-feeding flies, *Tabanus*, *Stomoxys*, and *Haematopota*, can transmit *T. evansi* [1]. Thailand has experienced *T. evansi* outbreaks over the past decades [19] in several animal hosts [2], with the prevalence of *Trypanosoma* spp. in cattle and buffalo from four regions of Thailand ranging from 0% to 7.48% [20,21,22,23,24,25]. However, *T. evansi* typing has never been conducted in Thailand. To fill this knowledge gap, this study first confirmed the *T. evansi* type using molecular characterization and then determined the prevalence and risk factor association with *T. evansi* infection in Thai small-scale livestock farms using an integrative control approach.

## 2. Materials and Methods

### 2.1. Sample Size Calculation

To estimate the prevalence of *Trypanozoon* infection in Thai livestock, the required sample size was calculated using the Scalex SP calculator [26], and blood samples were collected accordingly. The required sample size was 341, calculated with a margin of error (*d*) of ±2%, a 95% confidence level, and an adjustment of 10% for potential sample loss, as shown by the equation below:

$$n=\frac{Z^{2}P(1-P)}{d^{2}}$$

where *n* is the sample size, Z is the Z statistic for the level of confidence (1.96 for a 95% confidence level), and P is the expected prevalence of 3.29%, suggested by previous reports of the molecular prevalence of *Trypanozoon* in different regions of Thailand [20,21,22,23,24,25].

### 2.2. Study Design, Areas, Blood Samples, and Data Collection

#### 2.2.1. Study Areas

Stratified sampling was employed to estimate the true prevalence of *Trypanozoon* infection in Thai small-scale livestock farms with ≤60 animals in the Northern, Northeastern, Central, and Southern regions. Sixteen provinces were included, comprising Songkhla, Yala, Phatthalung, Nakhon Pathom, Ratchaburi, Chiang Rai, Phrae, Lampang, Tak, Nong Khai, Surin, Si Sa Ket, Sakon Nakhon, Ubon Ratchathani, Suphan Buri, and Kanchanaburi.

#### 2.2.2. Ethics Statement

The animal use protocol was reviewed and approved by the Institutional Review Board Statement of Approval for Kasetsart University Institutional Animal Care and Use Committee (Approval number: ACKU67-VET-083).

#### 2.2.3. Sample Collection

On each farm, 20% of the animals were sampled using convenience sampling to assess *Trypanozoon* prevalence. Blood samples were collected from cattle and buffalo via the jugular or tail vein and stored at −20 °C in the Department of Parasitology, Faculty of Veterinary Medicine, Kasetsart University, Bangkok, Thailand until used. Samples that tested positive for *Trypanozoon* during prevalence screening were subsequently included in molecular characterization.

Samples from seven horses and two dogs that were confirmed as *Trypanozoon*-positive were submitted to Kasetsart University Veterinary Teaching Animal Hospital, along with an in vitro cultured *T. evansi* Thai isolate TEDC 953 [27], and included in the molecular characterization.

#### 2.2.4. Data Collection

Data were collected through interviews with farmers using a questionnaire covering the types of animals, animal history, sex, age, breed, and clinical signs, as well as farm management, type of farming, disease history on the farm, presence of ectoparasites, and animal care [28].

### 2.3. DNA Extraction

DNA extraction was conducted using a commercial blood and tissue extraction kit following the manufacturer’s instructions (FavorPrep™, Ping Tung Biotechnology Park, Taiwan). The final elution volume was 50 µL, and the genomic DNA was stored at −20 °C in a freezer at the Department of Parasitology, Faculty of Veterinary Medicine, Kasetsart University, Bangkok, Thailand, until it was used as a DNA template.

### 2.4. Detection of Trypanozoon Using a Conventional PCR Targeting the ITS2 Gene

Conventional PCR with specific primers targeting the internal transcribed spacer 2 (ITS2) gene was conducted to confirm *Trypanozoon* infection in animals [29]. The reaction mixture contained 10 µL/reaction, containing 2.5 µL of Taq 2× master mix buffer (New England Biolabs, Ipswich, MA, USA), 0.2 µL of each primer (20 µM), 3.6 µL of distilled water (DW), and 1 µL of DNA template. The amplification conditions are described in Table 1.

### 2.5. Integrative Control Approach and Trypanozoon-Positive Farm Follow-Up

An integrative control approach was conducted by integrating the diagnostic services for vector-borne parasitic diseases and prevention of insect vector projects (FF (KU) 32.67) as previously described [31,32]. Project details were announced to the farmers via the selected Thai Department of Livestock Development (DLD) offices through official letters and phone calls. The diagnostic service was publicly available from June 2023 to June 2024. Small-scale livestock farmers who wished to participate registered by telephone, in person at provincial DLD offices, or via an electronic platform. Molecular diagnostic testing for vector-borne parasitic diseases was performed, and the results for each animal were reported to the farmer and local veterinarians within 14 days of sample collection. Infected animals were advised to be treated with 3.5–7 mg/kg of diminazene aceturate (Berenil^®^ R.T.U., Rahway, NJ, USA), following vector control and disease prevention, as described by Desquesnes et al. (2013a) [1]. These details were also communicated through an official document letter. Satisfactory evaluation and suggestions on the integrative control approach program were also conducted via interview and an electronic Google Form. *Trypanozoon*-positive farms were revisited, and animal blood samples were recollected for molecular detection of *Trypanozoon* and other blood parasites for evaluation of the integrative control approach.

### 2.6. Molecular Characterization of T. evansi and Nucleotide Sequencing

**Figure 1:**
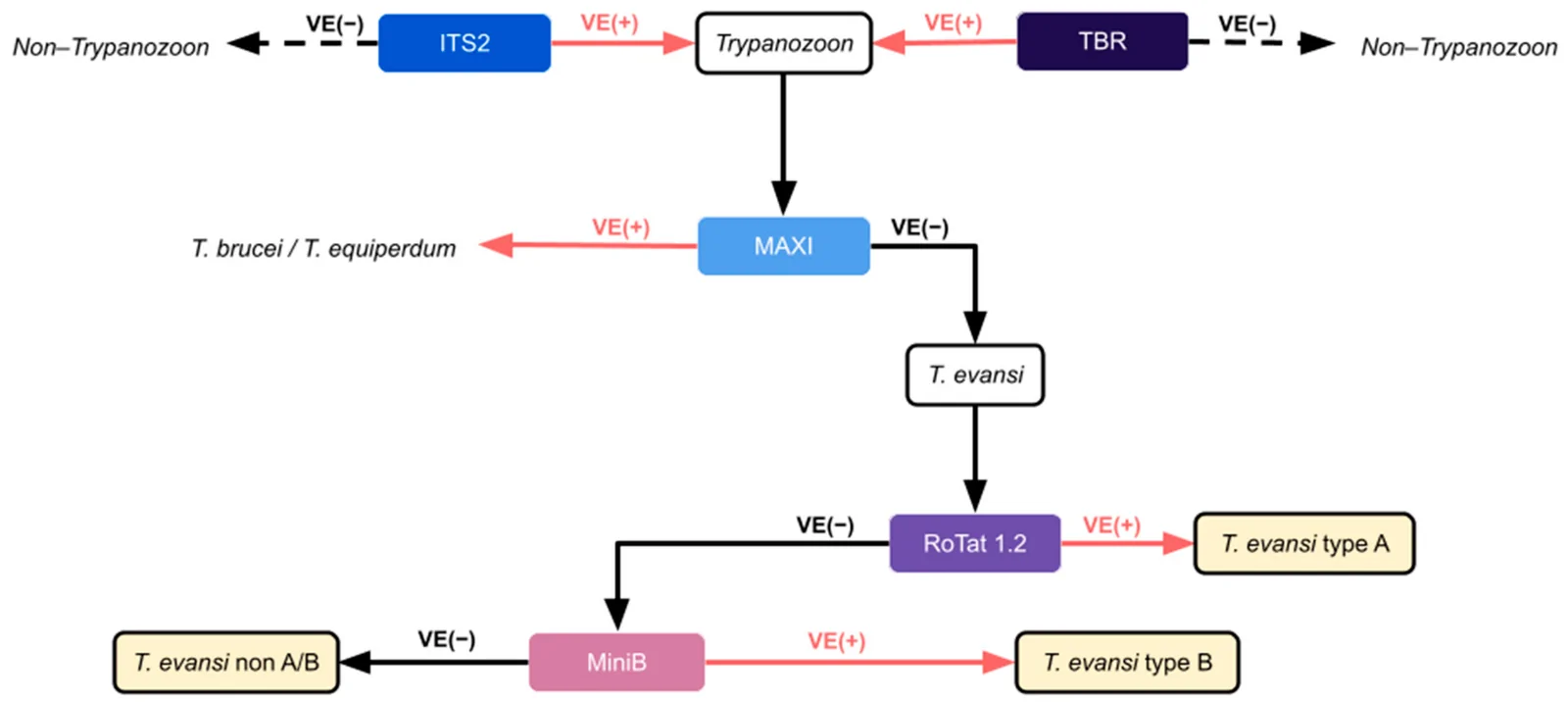
Molecular characterization of *T. evansi*. (Adapted from Subekti et al. (2024) [15]). The +ve = positive PCR test, −ve = negative PCR. Solid arrows indicate samples proceeding to the next PCR assay, while dashed arrows indicate exclusion from further testing. Pink arrows denote positive results, and black arrows denote negative results.

The molecular characterization of *T. evansi* was performed using four primer sets, including the TBR primer pair [12], which was used in accordance with the WOAH recommendation as the gold standard for the detection of *Trypanozoon* subgenus [11]. For TBR PCR, each reaction mixture consisted of 10 µL/reaction, comprising 1 µL of 10× PCR buffer, 0.6 µL of 25 mM MgCl_2_, 0.2 µL of 10 mM dNTP mix, 0.1 µL of each primer (20 µM), 0.05 µL of Platinum Taq DNA polymerase (Thermo Fisher Scientific, Waltham, MA, USA), 1 µL of DNA template, and 6.95 µL DW to adjust the final volume. *Trypanozoon* DNA stock with sequence confirmation was used as a positive control, while DW was used as a negative control. The ND5 primer pair [30] was used to exclude *T. brucei* and *T. equiperdum*. *Leishmania* DNA stock, with sequence confirmation used as a positive control, while DW was used as a negative control. The ILO7957/8091 primer pair [13] was used to confirm *T. evansi* type A. *T. evansi* type A DNA stock with sequence confirmation was used as a positive control, and DW was used as a negative control. The EVAB primer pair was used to confirm *T. evansi* type B [14]. *T. evansi* type B minicircle fragment synthesis was used as a positive control, and DW was used as a negative control. For ND5, ILO7957/8091, and EVAB PCR, each reaction mixture consisted of 10 µL/reaction, comprising 2.5 µL of Taq 2× master mix buffer (New England Biolabs, Ipswich, MA, USA), 0.2 µL of each primer (20 µM), 3.6 µL of DW, and 1 µL of DNA template. The PCR workflow was adapted from [15] and is shown in Figure 1. PCR amplification conditions for all primer sets used in this study are described in Table 1.

The PCR products were analyzed using electrophoresis on a 1.5% agarose gel (LE agarose, Thermo Fisher Scientific, Waltham, MA, USA) and visualized under gel documentation (Syngene, Cambridge, UK). The positive PCR products were excised from the gel and purified using NucleoSpin^®^ Gel and PCR Clean-up (MACHEREY-NAGEL GmbH & Co. KG, Düren, Germany). The PCR products were submitted for Sanger sequencing to confirm *Trypanozoon* species identification at U2Bio (Thailand) Co., Ltd., Bangkok, Thailand.

### 2.7. Bioinformatics and Phylogenetic Analysis

To confirm the *Trypanozoon* species, six nucleotide sequences derived from this study were also compared with previously published sequences in GenBank using the Basic Local Alignment Search Tool, version 2.17.0 (BLAST): https://blast.ncbi.nlm.nih.gov (accessed on 20 August 2025) in the National Center for Biotechnology Information (NCBI).

The phylogenetic tree was constructed based on the ITS2 gene sequences. Four sequences from this study and 35 sequences obtained from GenBank were aligned using Clustal W. The evolutionary history was analyzed using the neighbor-joining method. The percentage of replicate trees in which the associated taxa clustered together was evaluated using 1000 bootstrap replicates. The evolutionary distances were computed using the Maximum Composite Likelihood method within Molecular Evolutionary Genetics Analysis (MEGA) software version 11 [33].

### 2.8. Statistical and Risk Factors Analysis

The overall prevalence of *Trypanozoon* infection in cattle and buffalo was estimated from the ITS2 PCR as the percentage of positive results, with Chi-square (χ^2^) and Fisher’s exact tests used to select univariable factors at the individual level. Variables with *p* < 0.25 in the univariable analysis were considered candidates for inclusion in the logistic regression model [34]. A Generalized Linear Mixed Model (GLMM) was used to assess multivariable associations. Univariable and multivariable Firth’s penalized logistic regression were used to analyze data at the herd level. Variables with *p* < 0.05 and 95% confidence intervals were considered statistically significant risk factors associated with *Trypanozoon* infection in small-scale Thai livestock farms. Statistical analyses were performed using IBM SPSS Statistics, version 29.0.2.0 (IBM Corp., Armonk, NY, USA).

## 3. Results

### 3.1. Prevalence of Trypanozoon Infection in Thai Livestock

**Figure 2:**
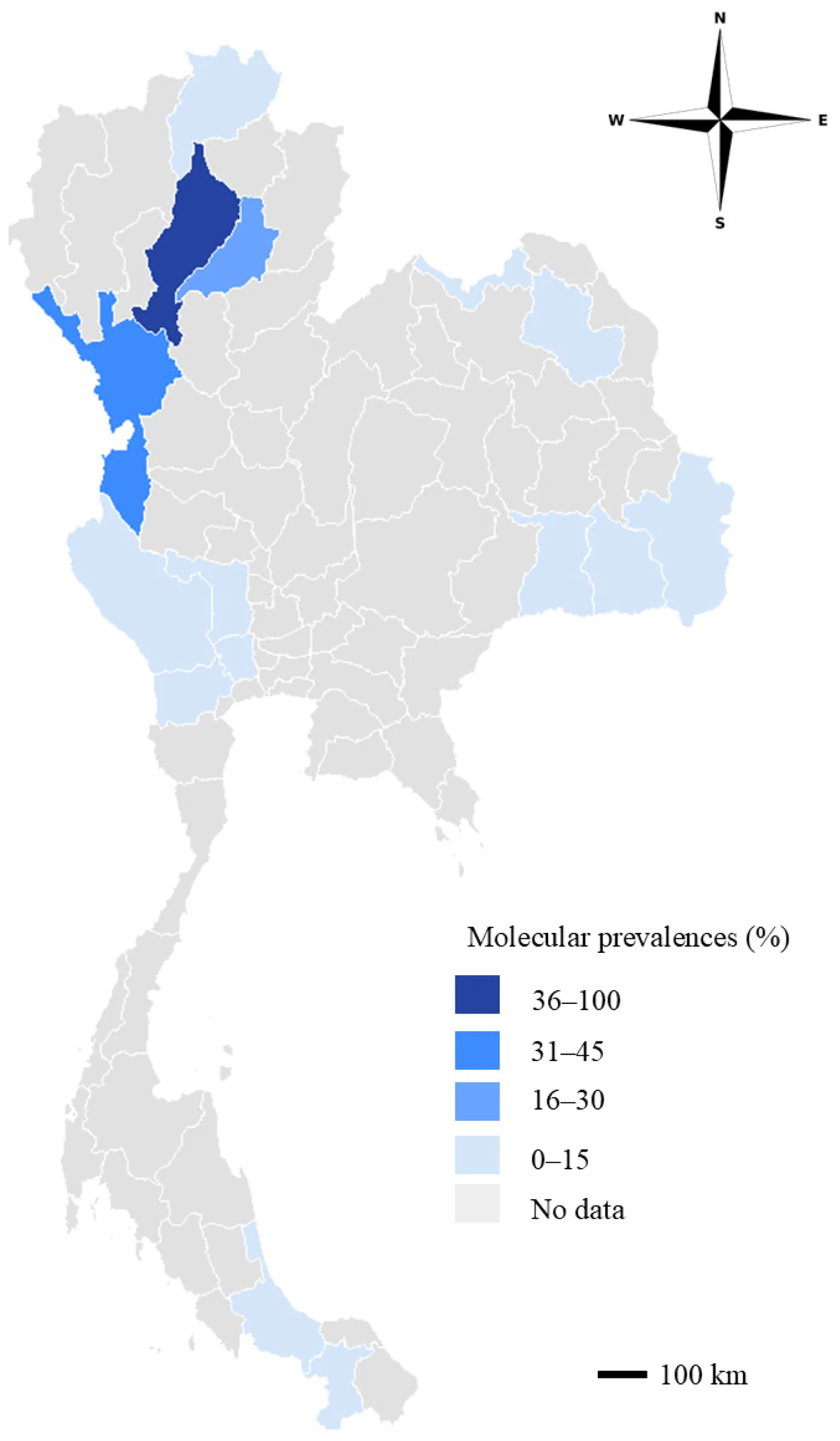
Distribution of *Trypanozoon* infection using molecular diagnosis of the ITS2 gene in cattle and buffalo across four regions of Thailand.

A total of 947 animals, including 374 beef cattle, 507 dairy cattle, 34 bullfight cattle, and 32 buffaloes from 123 farms across 16 provinces in 4 regions of Thailand, were included. The overall *Trypanozoon* prevalence was 3.17% (30/947) in Thai livestock. *Trypanozoon* prevalence was found predominantly in the Northern region at 15.30% (30/196), while no *Trypanozoon* infection in livestock was detected in the Northeastern, Central, and Southern regions (Table 2). All of the 30 *Trypanozoon*-positive samples were collected from beef cattle in Lampang (46.15%, 12/26), Tak (23.08%, 3/13), and Phrae (17.24%, 15/87) Provinces (Figure 2). *Trypanozoon* infection in beef cattle was 8.65% (30/374), with no *Trypanozoon* prevalence detected in dairy cattle, bullfight cattle, and buffalo. Among the 123 small-scale livestock farms, the 11 *Trypanozoon*-positive farms had at least one animal testing positive for the ITS2 gene.

### 3.2. Trypanozoon Was Negative After the Integrative Control Approach

Seven out of 11 farms that previously tested positive for *Trypanozoon* in Lampang and Phrae Provinces were revisited in June 2024, and 45 cattle blood samples were collected for *Trypanozoon* detection by PCR. No *Trypanozoon* was detected by PCR targeting the ITS2 gene in animals previously positive for *Trypanozoon*.

### 3.3. Molecular Characterization of Trypanozoon Species and T. evansi Typing in Thailand

Nucleotide sequences of TBR-positive samples from cattle (sample ID: P04) in the Northern region and from a horse (sample ID: HKPS02) were confirmed to belong to the *Trypanozoon* subgenus. BLAST (version 2.17.0) of the sequencing results showed 90.51–97.62% nucleotide identity with *T. evansi* (PQ373893) (Table 3). Nucleotide sequencing of the ITS2-positive samples showed that one cattle sample from Phrae Province (sample ID: P60), one horse (sample ID: HKPS04), and two dog samples (sample IDs: D01 and D02) were confirmed as *T. evansi* with 90.44% to 97.63% identity to the sequences published in GenBank (EF546001) (Table 3).

Among the 40 *Trypanozoon*-positive samples used for molecular characterization, none were positive for the NADH5 maxicircle kDNA (Table 4). Ten samples were positive with VSG RoTat 1.2, including one cattle (sample ID: P60), six horses (sample IDs: HKPS01, HKPS03-04, HKPS137-138, HKPS211), two dogs (sample IDs: D01 and D02), and one in vitro cultivation TEDC953 isolate. None of the samples tested positive for the minicircle B genes (Table 4).

### 3.4. Phylogenetic Analysis of T. evansi in Thailand

**Figure 3:**
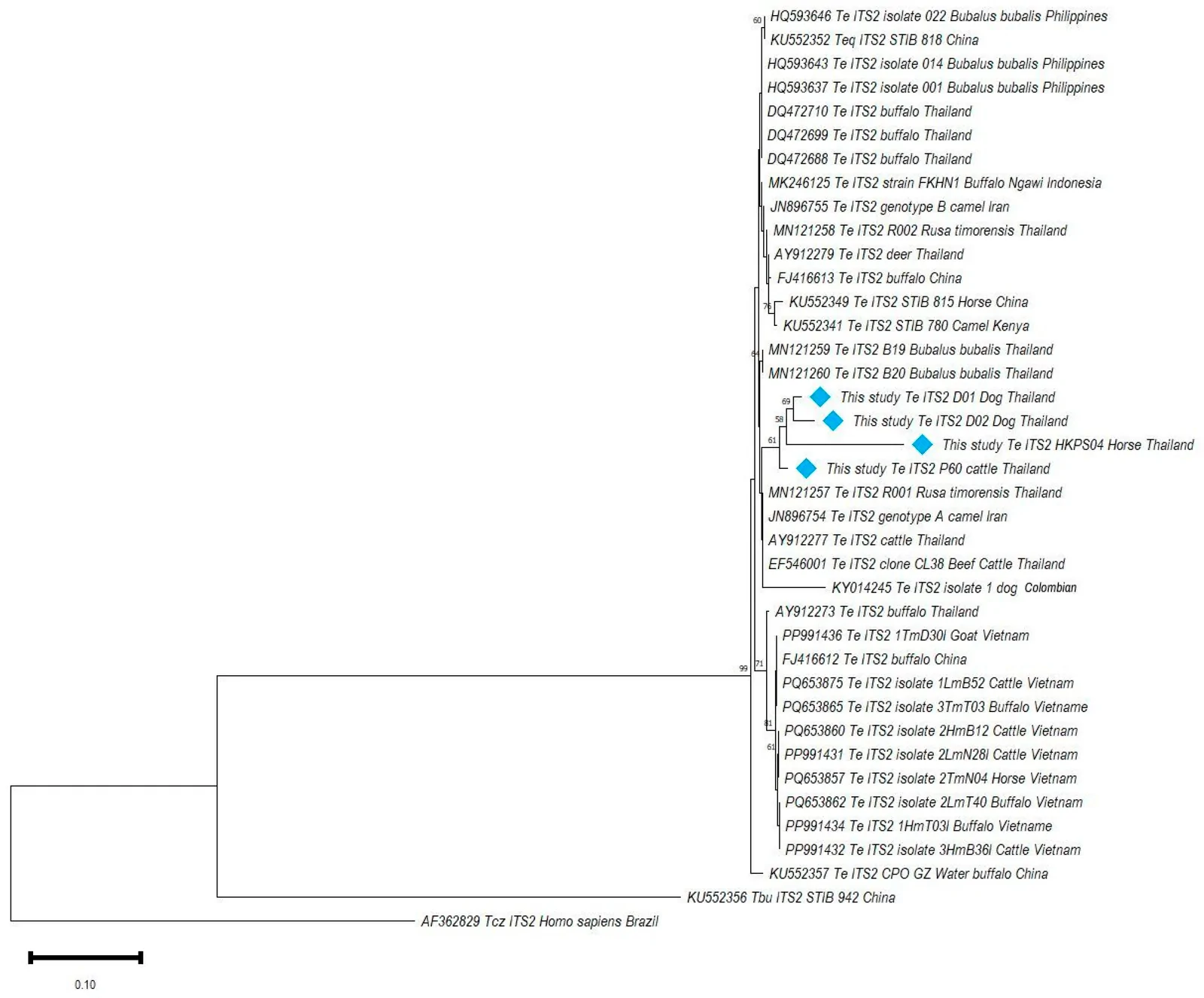
Phylogenetic relationships among *Trypanozoon* species, including *T. evansi*, 4 isolates from this study (blue rhombus), and 35 isolates from the GenBank database. The neighbor-joining tree was constructed using ITS2 sequences aligned with ClustalW and analyzed in MEGA 11. Evolutionary distances were computed using the Maximum Composite Likelihood method [33]. Bootstrap values (1000 replicates) >50% are indicated at nodes.

Phylogenetic analysis of the *Trypanozoon* ITS2 gene revealed that four samples P60 (PZ383266), D01 (PZ383267), D02 (PZ383268), and HKPS04 (PZ383270) were grouped in the main clade of *T. evansi* along with reference strains from Thailand and other countries, including *T. equiperdum*, which was clearly distinct from *T. cruzi* (AF362829) and *T. brucei* (KU552356), and supported by a bootstrap value of 99%. Within this subclade, the study samples showed a close relationship with *T. evansi* isolates previously reported in Thailand, including isolates from rusa deer (MN121257) and from cattle (AY912277), which also showed phylogenetic similarity with isolates from camels in Iran (*T. evansi* type A) and in dogs from Colombia (KY014245) (Figure 3).

### 3.5. Risk Factors Associated with Trypanozoon Infection in Thai Livestock

Univariate analyses at the individual level were conducted using Chi-square (χ^2^) and Fisher’s exact tests. Statistical significance (*p* < 0.25) was observed for associations of *Trypanozoon* infection with breed, sex, age, and region. Beef cattle exhibited the highest prevalence at 8.65% (30/374; *p* < 0.001), with no infections detected in dairy cattle (0/507), bullfighting cattle (0/34), or buffalo (0/32). Males showed higher infection rates at 63.64% (28/44) compared to females at 0.37% (2/537; *p* < 0.001). Infection prevalence decreased with increasing age (χ^2^ = 158.47; *p* < 0.001). Animals aged less than one year (<1 year) had the highest infection rate at 55.17% (16/29), followed by those aged 1–3 years at 5.48% (8/146), and animals aged more than three years (≥3 years) at 1.49% (6/403). Geographically, all infections were detected exclusively in the Northern region at 15.30% (30/196; *p* < 0.001), with no cases found in the Northeastern 0% (0/239), Central 0% (0/200), or Southern 0% (0/312) regions (Table 5).

GLMM analysis revealed that sex, age, and region were significantly associated with *Trypanozoon* infection. Male cattle had significantly higher odds of infection compared to females (OR: 24.27; 95% CI: 8.58–68.66; *p* < 0.001). Animals aged less than one year had significantly higher odds of infection (OR: 5.79; 95% CI: 1.47–22.69; *p* < 0.01), while animals aged 1–3 years had lower odds of infection (OR: 0.96; 95% CI: 0.34–2.68; *p* = 0.93) compared to animals aged more than three years. The Northern region had significantly higher odds of infection (OR: 5.55; 95% CI: 1.13–37.11; *p* = 0.03), with the Northeastern region showing lower risk of infection (OR: 0.86; 95% CI: 0.27–2.76; *p* = 0.81) (Table 5).

Data were collected on farm management practices from 122/123 farms (99.19% participation rate), with some questions left unanswered by farmers. Six factors were included in the univariable analysis, while the multivariable analysis (*p* < 0.25) included semi-grazing (farm type), weight loss, lacrimal (presence of clinical signs), quarantine of sick animals, *Tabanus* and *Stomoxys* (presence of ectoparasites) (Table 6). Out of the 123 farms, 11 (8.94%) had at least one infected animal. Across the farming systems, semi-grazing farms had the highest prevalence, 21.95% (9/41; *p* = 0.002), followed by stall barn systems at 2.74% (2/73), while free-grazing farms had no prevalence (0/7; *p* = 0.76). Among the clinical sign evaluated, lacrimal showed the highest prevalence at 36.36% (8/22; *p* = 0.24), followed by weight loss 13.95% (6/43; *p* = 0.16), hematuria 11.11% (2/18; *p* = 0.59), pale membranes 10.71% (3/28; *p* = 0.63), and lethargy 8.00% (5/50; *p* = 0.79), with the lowest observed in intermittent fever, 6.90% (2/29; *p* = 0.77). Farms with no history of trypanosomosis had the highest infection rate, 16.67% (10/60; *p* = 0.90), while farms with disease history exhibited a lower prevalence, 7.69% (1/13). No infections were detected among farms with an unknown history of trypanosomosis (0/6; *p* = 0.59). Farms that did not quarantine sick animals had a higher prevalence, 17.8% (5/28), than farms that implemented quarantine measures, 7.06% (6/85; *p* = 0.24). Farms with the presence of *Stomoxys* spp. showed a higher prevalence of infection, 15.79% (8/76; *p* = 0.04), followed by the presence of *Tabanus*, 13.89% (10/72; *p* = 0.04), and *Haematobia* (3/51; *p* = 0.25). No infections were detected on farms where no ectoparasites were observed (0%; 0/11; *p* = 0.42). Farms that did not support the prevention of ectoparasites exhibited the highest prevalence, 12.50% (2/16; *p* = 0.71), compared to farms that operated disease prevention procedures, 8.82% (9/102).

Multivariable analysis revealed that the presence of lacrimal animals and the presence of *Stomoxys* were significantly associated with *Trypanozoon* infection (*p* < 0.05) (Table 6). The presence of lacrimal animals exhibited significantly higher odds of infection (OR: 20.15; 95% CI: 3.19–253.57; *p* < 0.05), whereas weight loss in animals did not increase the odds of infection (OR: 0.54; 95% CI: 0.05–3.16; *p* = 0.51). The presence of *Stomoxys* on the farm was significantly associated with infection, with a 12 times higher risk (OR: 11.88; 95% CI: 1.06–1766.66; *p* = 0.04). The presence of *Tabanus* was associated with higher odds of infection, although this was not statistically significant (OR: 1.67; 95%CI: 0.25–18.17; *p* = 0.60). Semi-grazing farming had two times higher odds of infection (OR: 2.09; 95% CI: 0.34–13.70; *p* = 0.41) than stall barn, although this was not statistically significant. Quarantine implementation was associated with lower odds of *Trypanozoon* infection, although this was also not statistically significant (OR: 0.53; 95%CI: 0.10–2.81; *p* = 0.44).

## 4. Discussion

Trypanosomosis caused by *T. evansi* affects animal health and the economy in many countries [11]. In Thailand, *T. evansi* infection has been reported across a wide range of animals, including domestic animals such as dogs [1], and wildlife, including elephants [35] and deer [36]. Livestock animals are considered important reservoir hosts due to the chronic or asymptomatic nature of the infection [2]. Therefore, monitoring *T. evansi* infection status in Thailand, particularly regarding current infections, is vitally important to maintain both animal and public health.

In this study, the overall prevalence of *Trypanozoon* infection in Thai livestock was 3.17.%, lower than the prevalence reported in Indonesia (22.66%) [37]. The highest prevalence was observed in the Northern region (15.30%), with no *Trypanozoon* infection detected in the Central, Northeastern, and Southern regions. This finding contrasted with previous reports in Thailand. The prevalence observed in this study was higher than reported by Klinbumrung et al. (2024) [38], who recorded a prevalence of 0.9% in the Northern region. Focusing on each region, the prevalence in the Northeastern region was 0.45% [24], and higher than found in this study (0%). Similarly, the prevalence previously reported in the Central region of Thailand was 2.02 to 5.23% [21,25], and also higher than observed in this study. The absence of infection in the Southern region concurred with Kamyingkird et al. (2020) [23], who also reported zero prevalence. Differences between our study results and previous reports can be explained by the diverse geography, which impacted the environment and the abundance of insect vectors [39].

To follow up on the disease control program, farms where *Trypanozoon* infection was detected were revisited six months after the initial visit, and the farm owners were interviewed. Out of the 11 previously infected farms, seven were successfully revisited and all the animals were re-examined for *Trypanozoon* infection using PCR, including the 30 cattle that had previously tested positive and also all the additional cattle on the affected farms, with 45 cattle samples collected during the second visit. Notably, no samples tested positive for *Trypanozoon* infection by PCR. These results suggested that the integrative control approaches reduced the *Trypanozoon* infection burden in small-scale livestock farms in the Northern region, although the absence of a control group and incomplete farm follow-up limited definitive conclusions. This disease control program should be carefully monitored, and its effectiveness should be tracked in different regions to obtain clearer results.

The risk factor analysis suggested that sex, age, and region were significantly associated with *Trypanozoon* infection. Male animals had a significantly higher risk of infection than females, concurring with previous reports on beef cattle along the Thai-Myanmar border [25] and cattle from Nigeria [40]. Male cattle, particularly bulls, are commonly used for draft work, plowing, and transportation, potentially increasing their exposure to hematophagous insect vectors [41]. Physical stress associated with labor may also elevate cortisol levels, which in turn suppresses lymphocyte proliferation, cytokine production, and adaptive immunity [42].

Animals aged less than one year (<1 year) had a significantly higher risk of *T. evansi* infection compared with animals older than three years (≥3 years), concurring with a previously published study [25]. The lowest infection rate was observed in animals older than three years (≥3 years), consistent with Suwan et al. (2023) [24]. The higher prevalence in young animals was possibly related to their immature immune systems (e.g., lower IgG levels and reduced cytokine responses), as well as farmers generally administering trypanocidal treatment to older animals, resulting in increased susceptibility to infection in younger animals [43].

The Northern region showed a significantly higher risk of *T. evansi* infection, contradicting previous reports that indicated the highest prevalence in the Central region [44]. This higher prevalence in the Northern region was possibly linked to the greater diversity and abundance of insect vectors [45], influenced by meteorological conditions such as temperature and relative humidity, especially at the beginning of the rainy season [46]. The high prevalence in beef cattle was explained by management practices, as reported by Suwan et al. 2023 [24], where beef cattle are commonly used for labor and typically allowed to graze outdoors in farmyards. Consequently, these animals are at a higher risk of exposure to insect vectors.

The herd-level risk factor analysis determined only 11/123 positive farms, with Firth’s penalized logistic regression used to address bias and separation problems arising from the small number of positive farms and sparse data. The type of farming was not significantly associated with *Trypanozoon* infection. However, semi-grazing farming was associated with the highest infection rate among the farming systems examined, with two times higher odds of infection compared to stall barn farming. The semi-grazing system is commonly used on beef cattle farms [24]. The farmers allow their animals to graze outdoors or perform labor during the daytime until dusk, and they are housed in the barn at night. This finding concurred with a previous study of risk factors associated with *T evansi* infection in camels [47]. Outdoor grazing activities increase the risk of animal infections due to the chances of exposure to insect vectors, which might carry the parasite [2].

Among the clinical signs examined, only lacrimation was significantly associated with *Trypanozoon* infection in this study, contrasting with a previous report describing fever, anemia, emaciation, and edema as common clinical signs of *T. evansi* infection in cattle [48]. Lacrimal or ocular discharges in infected animals were linked to neurological signs when the parasite invaded the central nervous system and were commonly observed in buffalo [49]. Ocular discharges in cattle have previously been associated with *T. brucei* [50]. Animal weight loss was significant in the univariable analysis; however, multivariable analysis indicated that it had no statistical significance. Weight loss is a common clinical sign observed in bovines infected with *T. evansi* [2], but it can also be due to other blood parasite or helminthic infections [51], and the other clinical signs did not show a significant association.

The presence of *Stomoxys* ectoparasites on farms was associated with *T. evansi* infection, with *Tabanus* showing a two times higher risk of infection, although this association was not statistically significant. The 95% CI included the null value of 1, and the effect estimate was, therefore, considered inconclusive, possibly indicating increased risk, decreased risk, or no effect and not statistically significant [52]. However, the presence of biting flies showed a trend toward an association with *T. evansi* infection, concurring with Desquesnes et al. (2013a,b) [1,2], who identified *Tabanus* and *Stomoxys* as the main mechanical vectors of *T. evansi*.

Limitations of the statistical analysis were identified. First, the unequal sex distribution in the animal sample may have influenced the statistical analysis, resulting in a wide 95% CI range. Second, missing data required the exclusion of some animals from the analysis, which meant that the bullfighting cattle category and the Central region variable could not be included in the model. Third, clinical signs were collected at the farm level, whereby the presence of a clinical sign was recorded if at least one animal on the farm exhibited it, potentially reducing the precision of statistical analysis [53]. Future studies should address these limitations by adopting balanced sampling designs, complete data recording, and individual animal-level clinical data collection.

For the molecular characterization of *T. evansi* in this study, the ITS2 primer exhibited more positive results than the TBR primer in this study, while P60, HKPS4, D01, and D02 samples showed TBR(−ve)/ITS2(+ve) results. This finding contrasted with previous reports indicating that the TBR primer pair has higher sensitivity than other primers [54]. Two hypotheses were proposed to explain this result. The first posited a TBR primer mismatch. Van et al. (2021) [55] rediscovered heterogeneity among TBR sequences, categorized into the 177 bp TBR group and the 176 bp TBR group. The TBR primer pair [12], used in this study, was designed using 177-bp monomers. (K00392.1) showed a perfect match with the 176 bp TBR group but had 1–2 mismatches with the 177 bp TBR group. Analysis of TBR genotypes from 77 *Trypanozoon* strains revealed that *T. evansi* isolates from Asia were distributed across both the 177 bp and 176 bp TBR groups. Isolates from Indonesia and the Philippines belonged to the 177 bp TBR group [55], indicating that Asia supported variants of the TBR sequence. In Thailand, nucleotide sequences from *T. evansi* in different regions showed five different nucleotide positions [56], with mismatches between the primer and target sequences, especially at the 3′ end, resulting in amplification failure despite abundant templates [57]. The second hypothesis considered heme, breaking hemoglobin, and immunoglobulin G (IgG) as the main PCR inhibitors. Heme interferes with the function of DNA polymerase, reducing the efficiency of DNA amplification, while IgG competes for binding to a single DNA strand, preventing primers from binding and initiating the synthesis process [58]. Positive controls confirmed that the TBR(+ve)/ITS2(+ve) results reflected true differences in assay sensitivity, not technical artifacts. The TBR [12] and ITS2 primer pairs [29] provide genus-level detection by PCR; however, ITS2 can identify the species of parasite by nucleotide sequencing length, which varies considerably between species, but is generally conserved within the same species [59], while short sequences have high conservation and high copy number, but cannot identify species because they are too generic [11].

In this study, none of the samples showed positive results with the ND5 pair of primers [30], targeting the NADH dehydrogenase subunit 5 on maxicircle kDNA. This is not found in *T. evansi* due to the complete loss of maxicircle DNA [7]. These results indicated that no maxicircle kDNA was detected in *Trypanozoon* isolates, suggesting that neither *T. brucei* nor *T. equiperdum* was present among the samples in this study.

ILO7957/8091 primers targeting the VSG RoTat 1.2 gene [13] were used to determine *T. evansi* type A, and EVAB primers targeting the minicircle kDNA B gene were used to identify *T. evansi* type B. These two *T. evansi* types can be distinguished from each other by minicircle kDNA [60]. *T. evansi* type A is distributed worldwide, in Africa, South America, and Asia, with a wide host range [11], and has been reported in several animals including camel, cattle, horse, goat, and sheep. Type A has been reported in the Americas, Europe, Brazil, Peru, Bolivia, Venezuela, France, and Spain in procyonids, camels, horses, and dogs, and in Asia (The Philippines, Thailand, Indonesia, Malaysia, and India) in cattle, water buffaloes, and dogs [61]. By contrast, *T. evansi* type B has been reported in Africa, including Kenya, Sudan, Ethiopia, and Chad, where the *T. evansi* host range was limited to camels [62]. *T. evansi* type B was previously discovered as RoTat 1.2-negative isolates from Kenya [63]. Recently, *T. evansi* non-A/B, which was isolated from cattle and buffalo, has also been reported [15]. This isolate showed negative results with the ILO7957/8091 [13] and EVAB primers [14]. In this study, 10 samples were positive for the VSG RoTat 1.2 gene and negative for the minicircle B gene, which is found in horses, cattle, and dogs, while the in vitro cultivated isolate TEDC 953 was confirmed as *T. evansi* type A. However, 30 samples were negative for the VSG RoTat 1.2 gene and also for the minicircle B gene, and these were confirmed as *T. evansi* type non-A/B, which was only found in cattle. This study represents the second report of *T. evansi* non-A/B type in Southeast Asia, following its initial description in buffaloes and cattle from Indonesia [15]. The proportion of non-A/B isolates in this study was higher than that reported by Subekti et al. (2024) [15]. However, direct comparison between studies is limited by differences in host species, geographic origin, and sampling strategies. Further investigations are needed to determine whether this distribution pattern is representative of *T. evansi* type prevalence in Thai cattle.

In the Philippines, *T. evansi* isolates from water buffaloes displayed variable virulence and diminazene resistance profiles [17,18], highlighting that strain diversity within *T. evansi* can have significant clinical and management implications. Ethiopian *T. evansi* type A and type B isolates were sensitive to diminazene in both in vitro and in vivo studies, with no resistance observed [62]. However, susceptibility may vary depending on geographic origin and individual strain characteristics, rather than type classification alone. Whether the *T. evansi* type non-A/B, which was identified in this study, shares similar susceptibility to diminazene remains unknown and warrants further investigation.

A phylogenetic tree constructed using the ITS2 gene revealed that all the samples from this study were clustered in the main clade of *T. evansi* along with reference strains from Thailand and other countries, and clearly distinct from those of *T. cruzi* (AF362829) and *T. brucei* (KU552356) with 99% bootstrap support. The distinction between *T. brucei* and *T. evansi* using the ITS2 gene in this study concurred with a previous report that constructed a phylogenetic tree using the ITS1 and ITS2 genes. The ITS2 tree showed separately classified *T. brucei* and *T. evansi*, while the ITS1 tree showed that some isolates were within the same clade as *T. brucei* [64], with *T. equiperdum* clustered within the *T. evansi* clade. This result concurred with a previous study in Indonesia, which found that the ITS2 gene was unable to distinguish *T. equiperdum* (KU552352) from *T. evansi* [65]. However, the ITS2 gene successfully classified *T. evansi* isolates by geographic origin. The *T. evansi* isolates in this study were clustered within the same age as *T. evansi* isolates previously reported from Thailand and the Philippines, while the Vietnamese isolates were classified together and separated from the Philippines and Thailand, with bootstrap support of 71–81%. The similarity of the isolates between Thailand and the Philippines was due to the spread of pathogens through animal movement between the countries [66]. Bovines such as cattle and buffalo, which are considered reservoirs due to asymptomatic carriers, can induce *Trypanozoon* outbreaks [25]. In this study, samples P60, HKPS04, D01, and D02 demonstrated a close phylogenetic relationship with *T. evansi* isolates previously reported in Thailand from rusa deer (MN121257) and cattle (AY912277). Phylogenetic similarity was also shown with isolates from camels, *T. evansi* type A, and in dogs from Colombia (KY014245), which concurred with *T. evansi* type A in this study. The phylogenetic tree based on the ITS2 gene (Figure 3) shows an intermingling of host species that cannot be clearly classified. The short branch length, geographical classification, and intermixture of host species indicate that the same strain of *T. evansi* is circulating in the area. The successful use of the ITS2 gene to assess the diversity of *T. evansi* in this study underscores the benefits of ITS2 in studying the genetic diversity of *T. evansi*, consistent with previous studies both in Thailand [36,65] and abroad [66].

## 5. Conclusions

Our study results confirmed that *T. evansi*, type A and type non-A/B, continue to circulate among Thai livestock, with high infection rates in beef cattle in the Northern Lampang, Tak, and Phrae Provinces. Young age, male sex, farm-level lacrimation, and the presence of *Stomoxys* were identified as significant risk factors, highlighting the roles of host susceptibility, animal management, and vector exposure in disease transmission. Phylogenetic analysis based on the ITS2 gene demonstrated that the Thai isolates were closely related to previously reported *T. evansi* strains from Thai deer and cattle, as well as isolates from Iranian camels and Colombian dogs. Importantly, no *Trypanozoon* infection was detected on PCR re-examination of cattle at revisited farms, suggesting that the integrative control approach may help reduce the burden of trypanosomosis in small-scale livestock farms. The molecular and typing data generated in this study provide a foundation for future surveillance programs and the development of more effective diagnostic strategies to combat trypanosomosis in Thai livestock.

## Figures and Tables

**Table 1:** PCR conditions and primers used in this study.

Target Genes	Primers	Primer Sequences	Amplicon Length (bp)	Thermocycling Conditions	References
*Trypanosoma brucei* repeated region	TBR1	5′-GAA TAT TAA ACA ATG CGC AG-3′	164	94 °C for 1 min, 35 cycles of 94 °C for 30 s, 60 °C for 1 min, 72 °C for 30 s, and final extension at 72 °C for 2 min.	[12]
	TBR2	5′-CCA TTT ATT AGC TTT GTT GC-3′			
Internal Transcribed Spacer 2	ITS2F	5′-TGT CAC GCA TAT ACG TGT GTG-3′	347	95 °C for 2 min, 30 cycles of 95 °C for 30 s, 52.8 °C for 30 s, 72 °C for 30 s, and final extension at 72 °C for 2 min.	[29]
	ITS2R	5′-TAC ACA CAT ACA CAC TAT CCG-3′			
Maxicircle NADH 5	ND5-F	5′-TGG GTT TAT ATC AGG TTC ATT TAT G-3′	395	94 °C for 5 min, 30 cycles of 94 °C for 30 s, 51.2 °C for 30 s, 72 °C for 1 min, and final extension at 72 °C for 5 min.	[30]
	ND5-R	5′-CCC TAA TAA TCT CAT CCG CAG TAC G-3′			
VSG RoTat.1.2	ILO7957	5′-GCC ACC ACG GCG AAA GAC-3′	488	94 °C for 1 min, 35 cycles of 94 °C for 30 s, 60 °C for 1 min, 72 °C for 30 s, and final extension at 72 °C for 2 min.	[13]
	ILO8091	5′-TAA TCA GTG TGG TGT GC-3′			
Minicircle B	EVAB1	5′-CAC AGT CCG AGA GAT AGA G-3′	436	94 °C for 5 min, 30 cycles of 94 °C for 30 s, 60 °C for 30 s, and final extension at 72 °C for 1 min	[14]
	EVAB2	5′-CTG TAC TCT ACA TCT ACC TC-3′			

**Table 2:** Prevalence of *Trypanozoon* infection in livestock in each region of Thailand.

Regions	No of Tested Animals	Number of *Trypanozoon* Positive (ITS2 Gene)	Prevalence of *Trypanozoon* (%)
Northern	196	30	15.30%
Northeastern	239	0	0
Central	200	0	0
Southern	312	0	0
Overall	947	30	3.17%

**Table 3:** Sequencing and BLAST results of *Trypanozoon*-positive samples based on the TBR and ITS2 genes.

Sample ID	Sample Types	Hosts	Origins	TBR Gene	ITS2 Gene
				*T. evansi* (Reference Accession No: PQ373893)	*T. evansi* (Reference Accession No: EF546001)
P04	Infected Blood	Cattle	Phrae province, Thailand	97.62%	NA
P60	Infected Blood	Cattle	Phrae province, Thailand	NA	97.63%
HKPS02	Infected Blood	Horse	KU KPS animal hospital	90.51%	NA
HKPS04	Infected Blood	Horse	KU KPS animal hospital	NA	90.44%
D01	Infected Blood	Dog	KU animal hospital	NA	97.3%
D02	Infected Blood	Dog	KU KPS animal hospital	NA	95.41%

NA: Not Available.

**Table 4:** Molecular characterization of *Trypanozoon* and *T. evansi* typing in Thailand.

Order	Sample ID	Hosts	Origin	Gene Targets					*T. evansi* Type
				TBR	ITS2	Maxicircle NADH5	RoTat 1.2	Minicircle B	
1	P04	Cattle	Phrae province, Thailand	+ve	+ve	−ve	−ve	−ve	non A/B
2	P05	Cattle	Phrae province, Thailand	−ve	+ve	−ve	−ve	−ve	non A/B
3	P07	Cattle	Phrae province, Thailand	−ve	+ve	−ve	−ve	−ve	non A/B
4	P08	Cattle	Phrae province, Thailand	−ve	+ve	−ve	−ve	−ve	non A/B
5	P09	Cattle	Phrae province, Thailand	−ve	+ve	−ve	−ve	−ve	non A/B
6	P10	Cattle	Phrae province, Thailand	−ve	+ve	−ve	−ve	−ve	non A/B
7	P14	Cattle	Phrae province, Thailand	−ve	+ve	−ve	−ve	−ve	non A/B
8	P16	Cattle	Phrae province, Thailand	−ve	+ve	−ve	−ve	−ve	non A/B
9	P26	Cattle	Phrae province, Thailand	−ve	+ve	−ve	−ve	−ve	non A/B
10	P29	Cattle	Phrae province, Thailand	−ve	+ve	−ve	−ve	−ve	non A/B
11	P34	Cattle	Phrae province, Thailand	−ve	+ve	−ve	−ve	−ve	non A/B
12	P40	Cattle	Phrae province, Thailand	−ve	+ve	−ve	−ve	−ve	non A/B
13	P60	Cattle	Phrae province, Thailand	−ve	+ve	−ve	+ve	−ve	A
14	P77	Cattle	Phrae province, Thailand	−ve	+ve	−ve	−ve	−ve	non A/B
15	P78	Cattle	Phrae province, Thailand	−ve	+ve	−ve	−ve	−ve	non A/B
16	LP01	Cattle	Lampang province, Thailand	−ve	+ve	−ve	−ve	−ve	non A/B
17	LP02	Cattle	Lampang province, Thailand	−ve	+ve	−ve	−ve	−ve	non A/B
18	LP03	Cattle	Lampang province, Thailand	−ve	+ve	−ve	−ve	−ve	non A/B
19	LP04	Cattle	Lampang province, Thailand	−ve	+ve	−ve	−ve	−ve	non A/B
20	LP05	Cattle	Lampang province, Thailand	−ve	+ve	−ve	−ve	−ve	non A/B
21	LP06	Cattle	Lampang province, Thailand	−ve	+ve	−ve	−ve	−ve	non A/B
22	LP07	Cattle	Lampang province, Thailand	−ve	+ve	−ve	−ve	−ve	non A/B
23	LP08	Cattle	Lampang province, Thailand	−ve	+ve	−ve	−ve	−ve	non A/B
24	LP09	Cattle	Lampang province, Thailand	−ve	+ve	−ve	−ve	−ve	non A/B
25	LP10	Cattle	Lampang province, Thailand	−ve	+ve	−ve	−ve	−ve	non A/B
26	LP11	Cattle	Lampang province, Thailand	−ve	+ve	−ve	−ve	−ve	non A/B
27	LP12	Cattle	Lampang province, Thailand	−ve	+ve	−ve	−ve	−ve	non A/B
28	TK06	Cattle	Tak province, Thailand	−ve	+ve	−ve	−ve	−ve	non A/B
29	TK08	Cattle	Tak province, Thailand	−ve	+ve	−ve	−ve	−ve	non A/B
30	TK11	Cattle	Tak province, Thailand	−ve	+ve	−ve	−ve	−ve	non A/B
31	HKPS01	Horse	KU KPS animal hospital	+ve	−ve	−ve	+ve	−ve	A
32	HKPS02	Horse	KU KPS animal hospital	+ve	+ve	−ve	−ve	−ve	non A/B
33	HKPS03	Horse	KU KPS animal hospital	−ve	+ve	−ve	+ve	−ve	A
34	HKPS04	Horse	KU KPS animal hospital	−ve	+ve	−ve	+ve	−ve	A
35	HKPS137	Horse	KU KPS animal hospital	NA	+ve	−ve	+ve	−ve	A
36	HKPS138	Horse	KU KPS animal hospital	NA	+ve	−ve	+ve	−ve	A
37	HKPS211	Horse	KU KPS animal hospital	NA	+ve	−ve	+ve	−ve	A
38	D01	Dog	KU animal hospital	−ve	+ve	−ve	+ve	−ve	A
39	D02	Dog	KU KPS animal hospital	−ve	+ve	−ve	+ve	−ve	A
40	TEDC953	In vitro Cultivation	Parasitology Department VET KU	−ve	+ve	−ve	+ve	−ve	A

+ve = positive PCR test, −ve = negative PCR test, NA: not available.

**Table 5:** Risk factors associated with *Trypanozoon* infection in Thailand.

Factors	No. of Animals	*Trypanozoon*		Test	*p*-Value	GLMM	
		*n*	%			OR (95% CI)	*p*-Value
Breeds				Fisher Freeman Halton	<0.001 *		
Buffaloes	32	0	0			Ref.	
Beef Cattle	374	30	8.65			2.18 (0.25–19.18)	0.48
Dairy Cattle	507	0	0.00			4.62 (0.42–50.48)	0.21
Bullfighting cattle	34	0	0.00			NA	NA
Sex				Fisher’s exact	<0.001 *		
Female	537	2	0.37			Ref.	
Male	44	28	63.64			24.27 (8.58–68.66)	<0.001 **
Age				Chi–square	<0.001 *		
≥3 years	403	6	1.49			Ref.	
≥1–3 years	146	8	5.48			0.96 (0.34–2.68)	0.93
<1 year	29	16	55.17			5.79 (1.47–22.69)	0.01 **
Regions				Chi–square	<0.001 *		
Southern	312	0	0.00			Ref.	
Northern	196	30	15.30			5.55 (1.13–37.11)	0.03 **
Northeastern	239	0	0.00			0.86 (0.27–2.76)	0.81
Central	200	0	0.00			NA	NA

OR: odds ratio; CI: confidence interval; NA: not available; *: *p* < 0.25, **: *p* < 0.05.

**Table 6:** Farm management factors associated with *Trypanozoon* infection in Thailand (univariable and multivariable Firth’s penalized logistic regression).

Factors	No. of Farms	*Trypanozoon*		Univariable OR (95% CI)	*p*-Value	Multivariable Adjusted OR (95% CI)	*p*-Value
		*n*	%				
Type of farming							
Stall barn	71	2	2.8	Ref.		Ref.	
Semi-grazing	41	9	21.9	7.76 (2.01–42.74)	0.002 *	2.09 (0.34–13.70)	0.41
Free-grazing	7	0	0.00	0.651 (0.00–6.04)	0.76		
Presence of clinical signs							
Pale membranes	28	3	10.71	1.40 (0.33–4.93)	0.63		
Intermittent fever	29	2	6.90	0.81 (0.15–3.08)	0.77		
Weight loss	43	6	13.95	2.35 (0.69–8.16)	0.16 *	0.54 (0.05–3.16)	0.51
Hematuria	18	2	11.11	1.52 (0.27–6.03)	0.59		
Lethargy	50	5	8.00	0.85 (0.22–2.82)	0.79		
Lacrimal	22	8	36.36	16.33 (4.47–72.94)	0.24 *	20.15 (3.19–253.57)	<0.05 **
History of trypanosomosis on the farm							
No	60	10	16.67	Ref.			
Yes	13	1	7.69	0.90 (0.09–4.39)	0.90		
Don’t know	6	0	0.00	0.48 (0.00–4.46)	0.59		
Quarantine of the sick animals							
Not quarantine	28	5	17.8	Ref.		Ref.	
Quarantine	85	6	7.06	0.48 (0.14–1.68)	0.24 *	0.53 (0.10–2.81)	0.44
Presence of ectoparasites							
*Tabanus*	72	10	13.89	4.76 (1.06–45.26)	0.04 *	1.67 (0.25–18.17)	0.60
*Stomoxys*	76	11	14.47	8.57 (1.05–1112.94)	0.04 *	11.88 (1.06–1766.66)	0.04 **
*Haematobia*	51	3	5.88	0.48 (0.11–1.66)	0.25		
Absence of ectoparasite	11	0	0.00	0.35 (0.00–3.06)	0.42		
Prevention of ectoparasites				0.75 (0.19–4.17)	0.71		
Yes	102	9	8.82				
No	16	2	12.50				

OR: odds ratio; CI: confidence interval; NA: not available; *: *p* < 0.25, **: *p* < 0.05.

## Data Availability

The original data presented in the study are openly available in GenBank including, P60 accession number: PZ383266: https://www.ncbi.nlm.nih.gov/nuccore/PZ383266 (accessed on 8 May 2026), D01 accession number: PZ383267: https://www.ncbi.nlm.nih.gov/nuccore/PZ383267 (accessed on 8 May 2026), D02 accession number: PZ383268: https://www.ncbi.nlm.nih.gov/nuccore/PZ383268 (accessed on 8 May 2026), and HKPS04 accession number: PZ383270: https://www.ncbi.nlm.nih.gov/nuccore/PZ383270 (accessed on 8 May 2026).
